# The effect of the use of commercial tempeh starter on the diversity of *Rhizopus* tempeh in Indonesia

**DOI:** 10.1038/s41598-021-03308-6

**Published:** 2021-12-14

**Authors:** Wellyzar Sjamsuridzal, Mangunatun Khasanah, Rela Febriani, Yura Vebliza, Ariyanti Oetari, Iman Santoso, Indrawati Gandjar

**Affiliations:** 1 Department of Biology, Faculty of Mathematics and Natural Sciences, Universitas Indonesia, Depok, 16424 Indonesia; 2 Center of Excellence for Indigenous Biological Resources-Genome Studies, Faculty of Mathematics and Natural Sciences, Universitas Indonesia, Depok, 16424 Indonesia

**Keywords:** Evolution, Microbiology

## Abstract

At present, only a single *Rhizopus* species, *R. microsporus*, can be found in fresh tempeh produced in Java, Indonesia. The loss of diversity of *Rhizopus* in tempeh has been associated with the widespread use of commercial tempeh starter in Indonesia since the 2000s. However, the identities of the previous *Rhizopus* strains associated with tempeh, which have been preserved in a culture collection in Indonesia, have not been verified. The present study aimed to verify the identities of 22 *Rhizopus* strains isolated from tempeh produced using the traditional tempeh starters from the 1960s to the 2000s. Phylogenetic analysis based on the ITS regions in the rRNA gene sequence data, revealed that the *Rhizopus* strains belonged to the species *R. arrhizus* (five strains); *R. delemar* (14 strains); and *R. microsporus* (three strains)*.* Verification of the identities of these *Rhizopus* strains in the present study confirmed the loss of diversity of *Rhizopus* species in tempeh produced in Indonesia, particularly in Java. Our findings confirmed that the morphological changes in *Rhizopus* species isolated from tempeh as a result of centuries of domestication.

## Introduction

Tempeh is a soybean-based fermented food that is popular worldwide. It is regarded as a good source of protein and is easily digestible food. Tempeh has been a very popular traditional fermented food in Indonesia for many centuries that reported by Nout and Kiers in 2005^1^. It is a very important protein source in the Javanese diet^2^. The production of traditional tempeh is thought to have started in Indonesia in the early 1600s^3^. It originated from Central or East Java. English word tempeh comes from Indonesian “tempe”. The word “tempe” originated from Central Java, Indonesia. *Serat Centhini* is the first known manuscript in Java to mention this word “tempe”^3^. As written in “The History of Tempeh”^3^, traditionally since at least 1875, the name for this food in Indonesia was written *témpé*, with various accents being used. Then in August 1972, when Indonesia modernized its language, the accents were dropped and the word came to be spelled *tempe* (still pronounced TEM-pay). In English and other European languages, the word has come to be spelled “tempeh,” the final “h” being added to prevent the word from being pronounced “temp”. Hendrik Coenraad Prinsen Geerligs was the first European man who use the spelling *tempeh* in German article in 1896^3,4^. Van Veen and Schaefer in 1950^5^ were the first scientists used term *tempeh* in an English language article. Then Steinkrauss et al*.*^6^ were the first in the US. Since then, the word has consistently been spelled *tempeh* in European languages.

The taxonomy of the genus *Rhizopus* (Ehrenb. 1821) has undergone dramatic changes, especially in the last 40 years. It has been significantly changed from traditional^7–9^ to molecular approaches^10–14^. Since the description of *R. arrhizus* by Fischer in 1892 (Fischer 1892), hundreds of species have been described based on discrete morphological and physiological features^7,8^. In 1965, Inui et al*.*^7^ examined 449 *Rhizopus* species in their monographs. Almost 20 years later, Schipper^8^ and Schipper and Stalpers^9^ revised the classification of *Rhizopus* based on comprehensive morphological characters, temperature tolerance and mating. They separated the genus into three groups—*R. microsporus, R. stolonifer*, and *R. arrhizus* (= *oryzae*), with the re-integration of many species. Schipper^8^ synonymized 29 species with *R. arrhizus* (= *oryzae*). The group classification of Schipper^8^ and Schipper and Stalpers^9^ are widely accepted. In 1985, Ellis^15^ concluded that *R. arrhizus, Amylomyces rouxii,* and *R. delemar* are conspecific based on DNA renaturation experiments and proposed to accommodate them in three varieties.

Abe et al*.*^16^ established the first molecular phylogeny of *Rhizopus* based on three molecules of the ribosomal RNA-encoding DNA (rDNA) and confirmed the same taxonomic grouping *microsporus*-group, *stolonifer*-group, and *R. arrhizus*. Liu et al*.*^11^ combined analyses of the ITS regions of rRNA and *pyrG* genes data and only allowed eight species to be distinguished: *R. americanus, R. caespitosus, R. homothallicus, R. microsporus, R. reflexus, R. schipperae, R. sexualis,* and *R. stolonifer*. The remaining two morphologically distinct species, *R. arrhizus* and *R. niveus*, formed an unresolved cluster. They considered *A. rouxii* as synonymous of *R. arrhizus*. In the same year, Zheng et al.^12^ revised the monograph of *Rhizopus* and organized the genus into 10 species and seven varieties by reanalyzed the data from Liu et al*.*^11^ along with morphological data. Abe et al.^17^ used rDNA ITS, actin-1, and translation elongation factor 1a (TEF-1a) sequences to confirm the eight-species division of *Rhizopus*. They showed that the *R. microsporus* complex consisted of a single species. Dolatabadi^18^ investigated the species boundaries of *R. microsporus* using ITS, *ACT*, and *TEF* markers in combination with mating tests, morphology, physiology, ecology, geography, and MALDI-TOF MS data, and reduced the six varieties of *R. microsporus* (vars *microsporus*, *azygosporus*, *chinensis*, *oligosporus*, *rhizopodiformis,* and *tuberosus*; Liu et al*.*^11^) to synonyms. The widely held suggestion that the strains with the morphology of *R. oligosporus* represent a separate species that can be found just in fermented food sources i.e. tempeh should thus be rejected because positive mating results have been found between all varieties of *R. microsporus*, therefore all strains concluded as a single species, *R. microsporus*.

The species boundaries among *R. arrhizus* and *R. delemar* was studied by Abe et al.^10^ and Gryganskyi et al*.*^19^, they show clearly that *R. arrhizus* and *R. delemar* represent taxonomic entities that either deserves the rank of varieties or species. Dolatabadi et al.^13^ considered *R. arrhizus* consisted of two varieties, e.g. var. *arrhizus* and var. *delemar*, based on sequence data of multi-locus studies as well as amplified fragment length polymorphism (AFLP) and mating experiment. They found there is still zygospore formation between members of both varieties, although their number is reduced suggesting that the mating barrier is not complete yet. There is also a nomenclatural issue with *arrhizus*. *Rhizopus arrhizus* was described first, but *R. oryzae* has been used by most authors. Schipper^8^ treated *R. arrhizus* as a doubtful species. Ellis et al*.*^15^ took up the name *R. arrhizus* again by designating NRRL 1469 as *ex-neotype* strain of *R. arrhizus*. Zheng et al*.*^12^ in their monograph on *Rhizopus* preferred *R. arrhizus* over *R. oryzae*. Similarly, Dolatabadi et al*.*^13^ also use the name *R. arrhizus* based on the protologue of the first described *R. arrhizus*. Gryganskyi et al.^14^, also use the name *R. arrhizus* in their classification of the genus *Rhizopus* using phylogenomic approaches based on 192 orthologous genes. They classified *Rhizopu*s strains into four species, e.g. *R. microsporus, R. stolonifer, R. arrhizus,* and *R. delemar*. In the present study, we followed this classification system (taxonomy of *Rhizopus* sensu Gryganskyi et al.^14^).

Earlier studies on tempeh before the Second World War by Dutch microbiologists^3^ revealed that tempeh in Java was fermented with *R. arrhizus.* The first scientific report on tempeh was published in 1896 and was written by the Dutchman H.C. Prinsen Geerligs, who lived in Java^3,4^. He stated that tempeh was fermented by the mold *R. arrhizus*. The same species was also mentioned by van Veen and Schaefer^5^. Some reports around the 1960s^20^ also found that *R. arrhizus*, was the dominant species from highly preferred tempeh samples in Java, such as tempeh “Malang” and tempeh “Purwokerto”.

In the 1960s, the cottage-scale tempeh industry spread to all regions in Indonesia by using traditional methods for tempeh production and producing tempeh with various local tastes and flavors. The method for preparing the inoculum (tempeh starter) varied based on locality. In the traditional process, the previous batch of tempeh or the mold grown and dried on *Hibiscus tiliaceus* leaves (*daun Waru*) was used as the tempeh inoculum. These leaves are used to carry tempeh inoculum as natural starters (known as *usar* in Indonesia)*.* Following this, beans were wrapped using banana or other large leaves and finally placed in a warm location to ferment for 1 or 2 days^5,21^ Tempeh has a pleasant odor and a slight cheese-like flavor^6^. In the earlier study of tempeh by a group of scientists from Cornell University, USA, around the 1960s, revealed that *R. arrhizus* to be the essential microorganism isolated from Indonesian tempeh scrapings^6^.

The interest in tempeh produced in Indonesia rapidly increased among Indonesian scientists after the late 1960s. Several *Rhizopus* species associated with tempeh produced using the traditional process in Indonesia have been reported by Indonesian mycologists*.* Dwidjoseputro and Wolf^22^ reported *R. arrhizus*, *R. microsporus*, and *R. stolonifer* to be associated with tempeh and tempeh starters in Malang, Surakarta, and Jakarta.

Extensive research on tempeh was also conducted in the USA since the 1960s by groups of microbiologists and food scientists^2,5,6,23^. An Indonesian microbiologist, Ko Swan Djien from the Bandung Institute of Technology, West Java, brought tempeh samples from Java to the laboratory of Dr. Hesseltine at NRRL, USA, in 1961 in order to study tempeh fermentation^23^. Forty *Rhizopus* strains were isolated from these tempeh samples. These strains belonged to species: *R. achlamydosporus, R. arrhizus*, *R. formosaensis*, *R. microsporus* (= *R. oligosporus*), and *R. stolonifer*^23^*.* Hesseltine^23^ stated that only *R. arrhizus* and *R. microsporus* (= *R. oligosporus*) were commonly used to produce tempeh. Wang and Hesseltine^24^ reported the best strain for producing tempeh from wheat and soybeans was *R. microsporus* (= *R. oligosporus*) NRRL 2710. Since they claimed that *R. microsporus* (= *R. oligosporus*) as the best tempeh mould, this species was then used by many Indonesian microbiologists for their study on tempeh (Gandjar and Santoso)^20^.

Large-scale commercial tempeh production began in the 1980s with the aim of guaranteeing a good tempeh product. The first commercial inoculum for tempeh, which consisted of mixed cultures of *R. arrhizus* and *R. microsporus*, was developed by the Chemistry Institute-Indonesian Institute of Sciences (LKN-LIPI) and the Cooperative of Tempeh and Tofu Producers of Indonesia (KOPTI) in 1985; they then distributed it to tempeh producers^20^. The next generation of commercial tempeh starter developed by LIPI was *Raprima*, containing only a single species, *R. microsporus. Raprima* has been produced by PT. Aneka Fermentasi Industri, Bandung, Indonesia, since 2001, and is widely used in tempeh fermentation in Indonesia and abroad.

Taxonomy of *Rhizopus* strains obtained from tempeh in Indonesia has been well studied by many scientists in abroad and those strains are well maintained at Centraalbureau voor Schimmelcultures KNAW (currently hosted by Westerdijk Institute) (The Netherlands), others in Mycothèque de l'Université catholique de Louvain (MUCL) (Belgium) and Northern Regional Research Laboratory (NRRL) (USA). On the other side, it is difficult to trace the genetic diversity of *Rhizopus* spp. previously used for tempeh production that preserved in culture collections in Indonesia, because *Rhizopus* cultures were rarely collected or were never preserved properly in culture collections in Indonesia. Their representation within sequence database is lacking and their molecular study has never been reported.

One of the authors (I. G.), collected *Rhizopus* strains and accumulated hundreds of strains from almost all regions in Indonesia since the 1960s. These *Rhizopus* strains have been preserved in the Universitas Indonesia Culture Collection (UICC), Depok, Indonesia. It is the only culture collection in Indonesia that maintains the *Rhizopus* strains isolated from tempeh produced using the traditional tempeh starters. Because of a lack of budget, this collection of *Rhizopus* strains was originally maintained only as living cultures; therefore, many strains have been lost. Since 2012, the strains have been maintained using a long-term preservation method, the liquid drying (L-drying) method, after financial support was obtained from the Society for Applied Microbiology of the United Kingdom (SfAM UK) Endangered Collection Grant.

At present, only 127 *Rhizopus* strains available from those isolated from tempeh produced using traditional starters (1960s–2000s) that are preserved in UICC. The molecular identification of these strains was not performed until 2017, when we sequenced 15 strains of *Rhizopus* from UICC based on the ITS regions of ribosomal RNA (rRNA) gene^25–27^. The present study aimed to sequence another 22 strains the *Rhizopus* strains from UICC based on the ITS regions of ribosomal RNA (rRNA) gene, to provide the accurate taxonomic identity of *Rhizopus* strains that were isolated from tempeh produced using traditional tempeh starters (1960s–2000s).

## Materials and methods

### Fungal strains and preservation methods

All *Rhizopus* strains were obtained from UICC, Center of Excellence for Indigenous Biological Resources-Genome Studies, FMIPA Universitas Indonesia, Depok, Indonesia. UICC maintains 127 *Rhizopus* spp. strains that originated from various types of tempeh (e.g., *tempe kedelai, tempe gembus, tempe kopra, tempe kedelai hitam, tempe koro, tempe koro wedus, tempe benguk, tempe kapok,* and *tempe lamtoro*) and traditional tempeh starters (e.g. *laru daun waru* and *laru daun pisang*) and were isolated from the 1960s to the 2000s. The origin of the 22 strains used in the present study and their year of isolation are provided in Table 1. Tempeh and tempeh starter samples were obtained from many regions in Indonesia, particularly those in Java, Kalimantan, Nusa Tenggara, Papua, Sulawesi, and Sumatera. The regions were as follows: Java (Jakarta, Cilacap, Magelang, Malang, Pacitan, Salatiga, Semarang, Solo, Surabaya, Tegal, Wonogiri, Wonosari, and Yogyakarta), Kalimantan (Balikpapan and Palangkaraya), Nusa Tenggara (Mataram), Papua (Wamena), Sulawesi (Manado), and Sumatera (Banda Aceh). Three samples per place of tempeh were collected. Long-term preservation of the cultures was performed using Liquid-drying method in lyophilized tubes and in glycerol solution (at − 80 °C).

**Table 1:** List of *Rhizopus* spp. strains collection isolated from tempeh starter (inocula) and tempeh used in this study.

No	Strain code	Species identity based on morphology and physiology	Location, source	Year of isolation	DDBJ accession number	Species Identity based on ITS rRNA gene	BLAST Homology sequence (%)
1	UICC 1	*R. oryzae*	Surabaya, *tempe gembus*	1971	LC514296	*R. delemar*	654/655 (99%)
2	UICC 3	*R. oligosporus*	Salatiga, *tempe gembus*	1971	LC514297	*R. delemar*	622/628 (99%)
3	UICC 4	*R. oryzae/R. chlamydosporus*	Salatiga, *tempe gembus*	1971	LC514298	*R. delemar*	653/654 (99%)
4	UICC 8	*R. cohnii*	Salatiga, *tempe gembus*	1972	LC514299	*R. arrhizus*	630/631 (99%)
5	UICC 9	*R. arrhizus*/*R. microsporus*	Tegal, *tempe gembus*	1973	LC514300	*R. delemar*	654/654 (100%)
6	UICC 11	*R. arrhizus/R. oligosporus*	Semarang, *tempe gembus*	1973	LC514302	*R. arrhizus*	652/652 (99%)
7	UICC 12	*R. oryzae*/*R. oligosporus*	Malang, *tempe kedelai*	1962	LC514303	*R. delemar*	652/654 (100%)
8	UICC 13	*R. oligosporus*	Cilacap, *tempe gembus*	1972	LC514304	*R. delemar*	654/656 (99%)
9	UICC 17	*R. oligosporus*	Yogyakarta, *tempe gembus*	1972	LC514305	*R. delemar*	650/655 (99%)
10	UICC 21	*R. microsporus* var. *chinensis*	Yogyakarta, *tempe gembus;*	1973	LC514306	*R. delemar*	624/628 (99%)
11	UICC 24	*R. oligosporus*	Yogyakarta, *tempe gembus*	1972	LC514307	*R. delemar*	653/656 (99%)
12	UICC 28	*R. arrhizus*	Yogyakarta, *tempe gembus*	1972	LC514310	*R. arrhizus*	652/652 (100%)
13	UICC 38	*R. cohnii*	Malang, *tempe kedelai*	1962	LC514315	*R. arrhizus*	586/587 (99%)
14	UICC 42	*R. oligosporus*	Yogyakarta, *tempe koro*	1972	LC514318	*R. delemar*	651/656 (99%)
15	UICC 53	*R. microsporus* var. o*ligosporus*	Yogyakarta, *tempe benguk*	1972	LC514320	*R. delemar*	624/629 (99%)
16	UICC 124	*R. arrhizus*	Magelang, *tempe benguk*	1974	LC514328	*R. delemar*	652/655 (99%)
17	UICC 500	*R. microsporus* var. *chinensis*	Aceh, *tempe kedelai*	1996	LC514330	*R. microsporus*	697/699 (99%)
18	UICC 520	*R. microsporus var. rhizopodiformis*	Manado, *daun waru*	1996	LC514331	*R. delemar*	631/631 (100%)
19	UICC 524	*R. oryzae*/*R. arrhizus*	Wamena, *tempe kedelai*	1998	LC514332	*R. delemar*	654/655 (99%)
20	UICC 531	*R. microsporus* var*. microsporus*	Balikpapan, *tempe kedelai*	2003	LC514333	*R. microsporus*	698/699 (99%)
21	UICC 536	*R. arrhizus*	Palangkaraya, *tempe kedelai*	2003	LC514334	*R. arrhizus*	650/653 (99%)
22	UICC 539	*R. microsporus* var. *oligosporus*	Mataram, *tempe kedelai*	2003	LC514335	*R. microsporus*	699/699 (100%)

Molecular identification results of *Rhizopus* spp. strains collection of UICC based on ITS region of rRNA gene sequence data and their DDBJ accession number.

### Fungal growth medium

Potato dextrose agar (PDA, Difco) was used as the growth medium for stock cultures and working cultures, for purifying the cultures, and for preparing DNA isolation, while 4% malt extract (Acumedia) agar (Difco) (MEA 4%) was used as the growth medium for morphological characterization. Macroscopic and microscopic observations of colonies, size and shape of spores were performed using a microscope [ZEISS Primostar Axio-Cam]. Monographs of *Rhizopus* were used as references for comparing the morphological data^8,9,12^.

### Fungal identification

The extraction of fungal genomic DNA was performed using the PrepMan™ Ultra Kit (Applied Biosystems, Foster City, CA) as described previously^25–27^. The ITS regions in the fungal rRNA gene were amplified using ITS universal fungal primers, namely ITS1 (5′-TCCGTAGGTGAACCTGCGG-3′) and ITS4 (5′-TCCTCCGCTTATTGATATGC-3′)^28^. PCR was performed under the following conditions: 95 °C for 1 min; 40 cycles at 94 °C for 1 min; 60 °C for 1 min; and 72 °C for 1 min; and a final extension cycle at 72 °C for 5 min. The PCR product was purified with a QIAquick Purification Kit (Qiagen). For sequencing of the ITS regions, the primers ITS5 and ITS4 were used)^28^. Sequencing reactions were conducted using a BigDye Terminator v3.1 Cycle Sequencing Ready Reaction Kit (Applied Biosystems) Foster City, CA, USA) following the manufacturer’s instructions. The gel electrophoresis and data collection were performed on ABI Prism 310 Genetic Analyzer (Applied Biosystems), or the PCR products of the ITS regions of rDNA were sent to 1st BASE (Malaysia) for sequencing. The fungal strains were identified according to their sequence homology with fungal sequences obtained from the GenBank DNA database hosted by NCBI (http://blast.ncbi.nlm.nih.gov) using the BLAST search tool^29^.

### Phylogenetic analyses

Sequence assembly and editing were performed using ChromasPro ver.1.7.7, while sequence alignment and phylogenetic tree construction were performed using ClustalX and MEGA 7, respectively^30,31^. Phylogenetic trees were constructed using the neighbor-joining (NJ)^32^, minimum evolution (ME)^33^, and maximum likelihood (ML)^34^ methods with 1000 bootstrap replications^35^. Evolutionary distances in the NJ method were computed using the Kimura 2-parameter method^36^. *Phycomyces blakesleeanus* NBRC 5823 was used as an outgroup. The identity of each fungal strain to the species level was verified according to the currently described species concept of the genus *Rhizopus*^28^. The ITS rRNA gene sequence accession numbers of the *Rhizopus* strains identified in the present study (LC514296–LC514335) have been deposited in the DNA Database of Japan (DDBJ, https://www.ddbj.nig.ac.jp) (Table 1).

## Results

### Re-identification of *Rhizopus* strains isolated from tempeh

A homology search was performed with the BLAST tool in DDBJ using the ITS regions in the rRNA gene sequence data of the fungal strains as a query; the results indicated that all the 22 strains in this study had very high homology (99–100%) to their closest species (Table 1).

### Phylogenetic analyses

**Figure 1:**
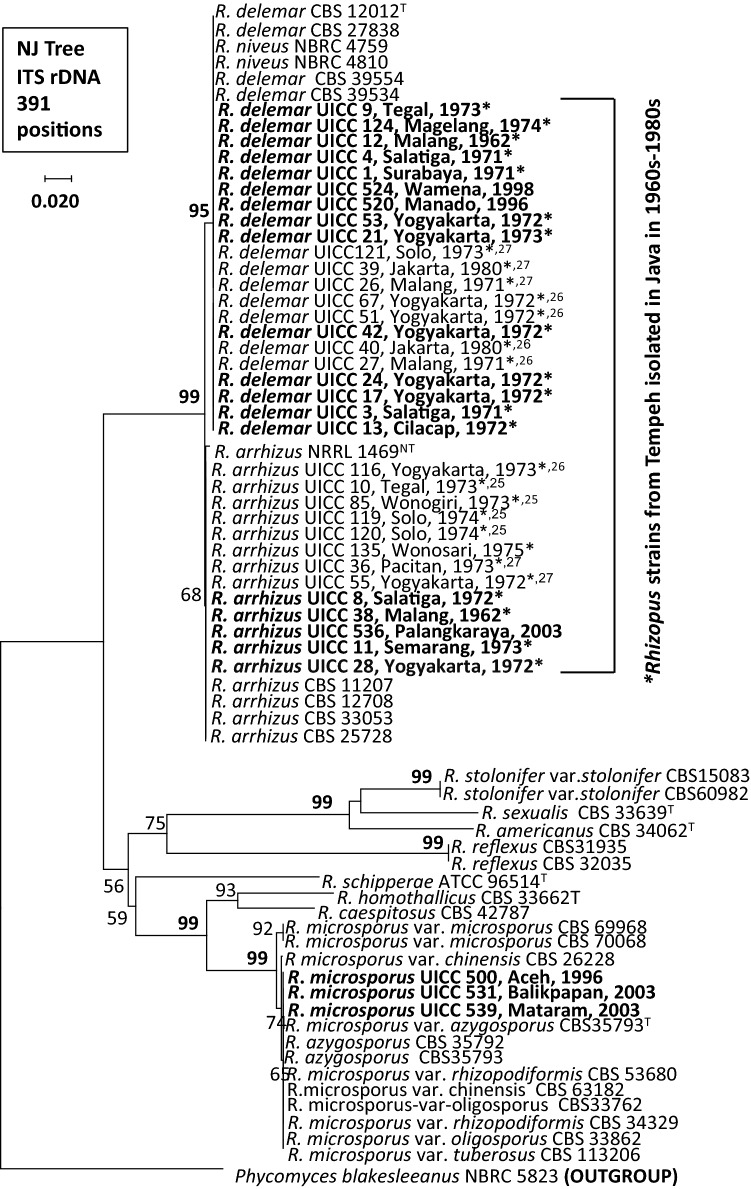
Phylogenetic tree of 37 *Rhizopus* strains from tempeh based on ITS rRNA gene sequence data: 22 strains determined in this study (indicated in bold face) and 15 strains from our previous works^25–27^. Asterisks (*) indicated *Rhizopus* strains from tempeh isolated in Java in 1960s–1980s. The tree was constructed using the NJ method^32^. Bootstrap values less than 50% are not shown.

Phylogenetic analyses using the NJ method and two other methods (ME and ML methods; data not shown) based on 70 OTUs revealed that all members of the genus *Rhizopus* were grouped into three major clades: *R. arrhizus*–*R. delemar, R. microsporus,* and *R. stolonifer* clades (Fig. 1). Analysis of the phylogenetic tree consisting of 22 strains determined in this study and 15 strains from our previous studies^25–27^, revealed that the strains isolated in the different regions in Indonesia before the commercial tempeh starters have been widely used in Indonesia belonged to three species: *R. arrhizus* (five strains), *R. delemar* (14 strains), and *R. microsporus* (three strains). All the strains isolated from Java in the 1960s–1970s belonged to *R. arrhizus* and *R. delemar.* The other strains isolated outside Java in 1996–2003 (Aceh, Balikpapan, Manado, Mataram, Palangkaraya, and Wamena) belonged to *R. arrhizus, R. delemar,* and *R. microsporus*.

### Morphological characteristics

**Figure 2:**
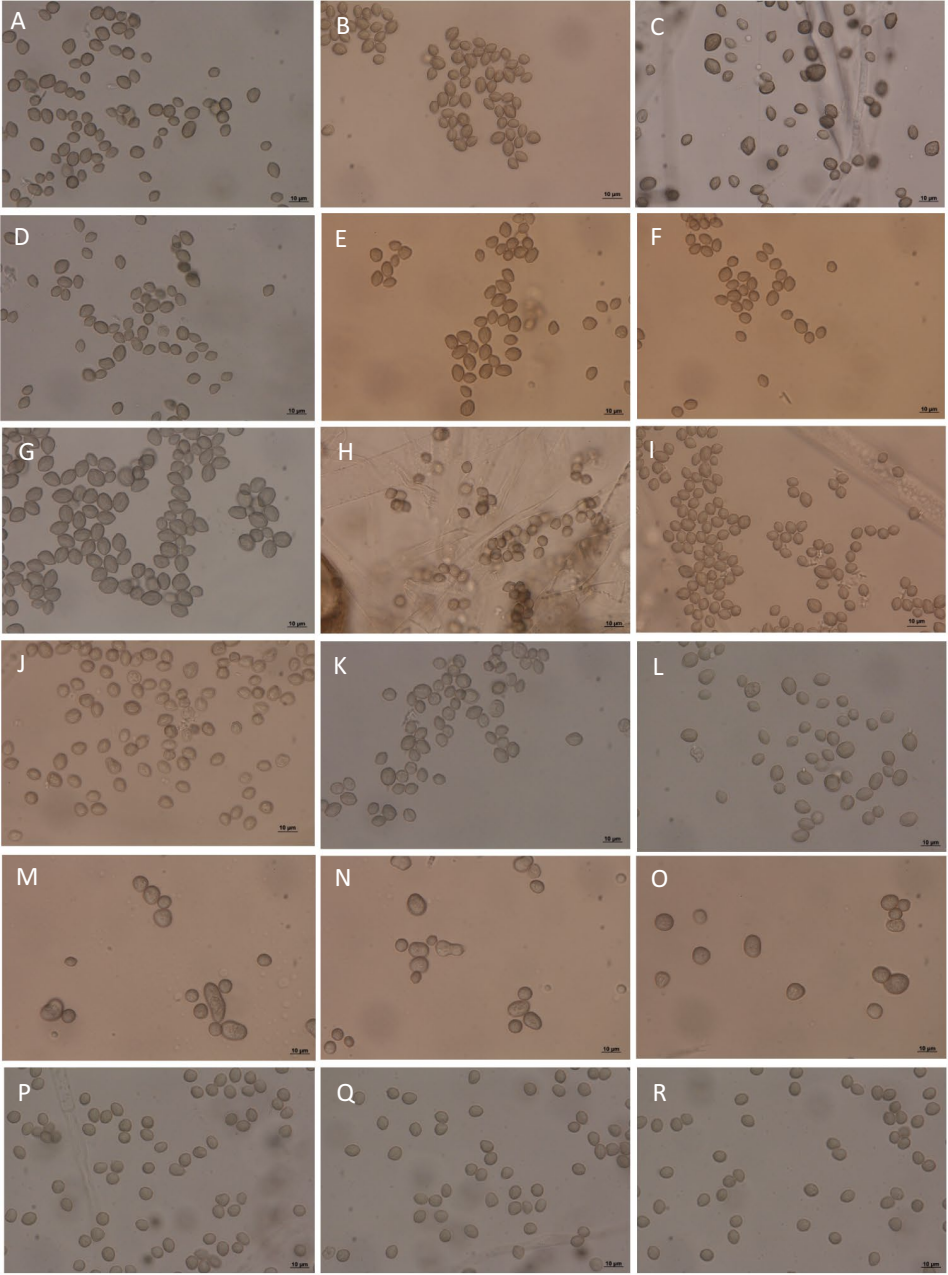
Sporangiospores of *Rhizopus* from tempeh as seen under light microscope. (**A**–**F**) *R. arrhizus* UICC 10, UICC 36, UICC 85, UICC 116, UICC 119, UICC 135; sporangiospores are angular, globose, subglobose, and irregular, striated. (**G**–**L**) *R. delemar* UICC 121, UICC 67, UICC 27, UICC 26, UICC 40, UICC 39, sporangiospores are angular, globose, subglobose, and irregular, striated. (**M**–**R**) *R. microsporus* UICC 500, UICC 531, sporangiospores are globose to subglobose, some are large and irregular, smooth. Three days on 4% MEA. Bars = 10 μm.

Morphological characterization was performed after 3 days incubation on 4% MEA to confirm the identities of the strains based on molecular identification. Light microscopic examination showed that sporangiospores of *R. arrhizus* (UICC 10, UICC 36, UICC 85, UICC 116, UICC 119, UICC 135) are angular, globose, subglobose, and irregular, striated, up to 8 µm in length; *R. delemar* (UICC 121, UICC 67, UICC 27, UICC 26, UICC 40, UICC 39) are angular, globose, subglobose, and irregular, striated, up to 8 µm in length; and *R. microsporus* (UICC 500, UICC 531) are globose to subglobose, some are large and irregular, smooth, up to 9 µm maximum diameter (Fig. 2).

**Figure 3:**
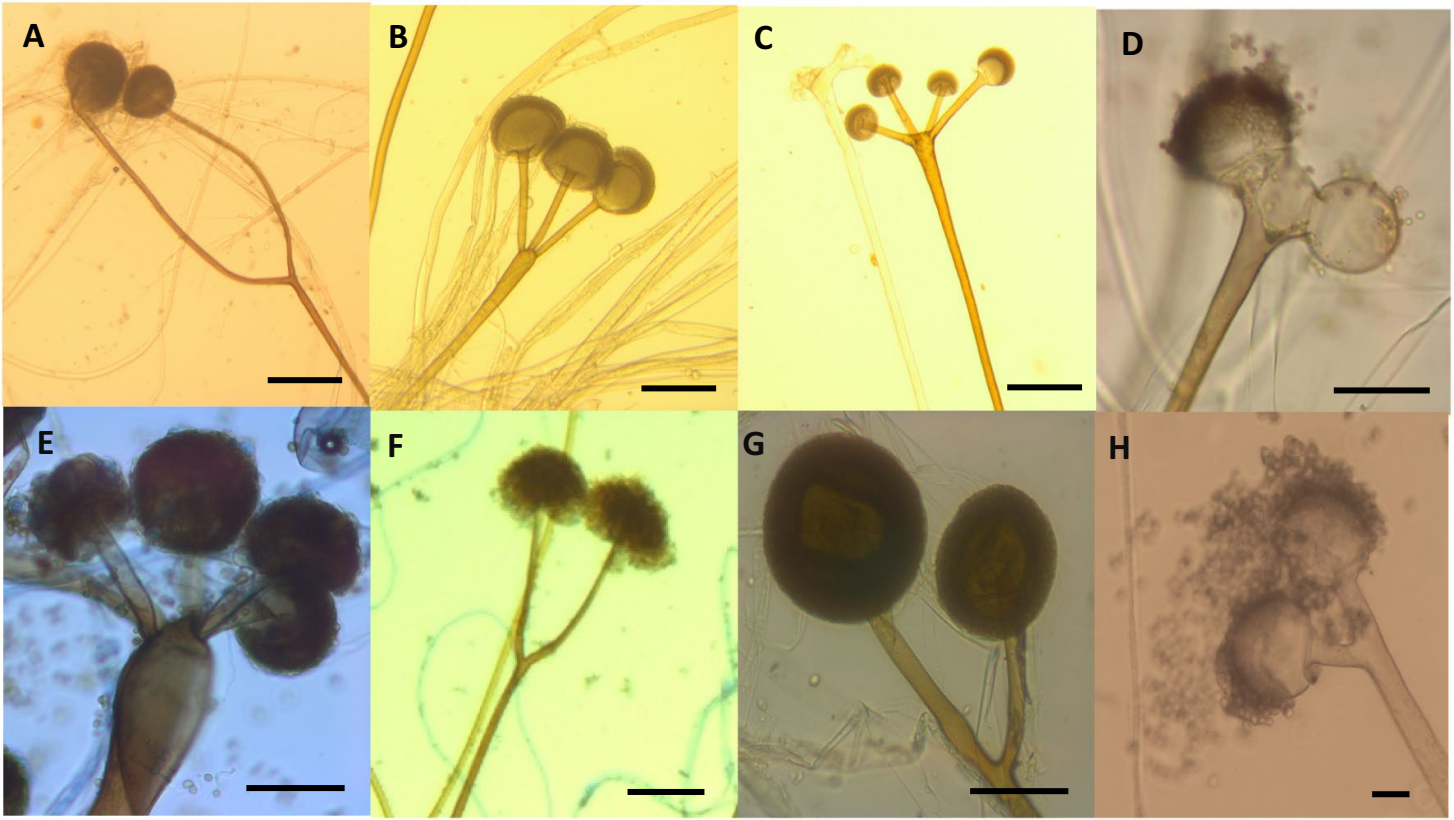
Branching sporangiophores with multi-sporangia of *Rhizopus* from tempeh: (**A**,**B**) *R. arrhizus* UICC 36, UICC 120, (**C**,**D**) UICC 10, (**E**) UICC 119; (**F**,**G** *R. delemar* UICC 40, UICC 26; (**H**) *R. microsporus* UICC 539. Seven days on 4% MEA. (**G**) photo credit to Vebliza^47^. Scale bar = 10 μm.

Light microscopic examination revealed a peculiar form of sporangiophores in *Rhizopus* strains. *Rhizopus arrhizus, R. delemar,* and *R. microsporus* strains possessed sporangiophores with unique morphological characteristics. These sporangiophores showed swelling and branching or were sometimes forked at the middle, upper-half, or apical position. The number of sporangia in a single sporangiophore varied between two and six, and they were arranged in a verticillate pattern (Fig. 3).

## Discussion

In the present study, we accurately identified the *Rhizopus* strains from tempeh isolated from traditional starters, before the widespread used of commercial tempeh starter in Indonesia. Based on the current taxonomy of *Rhizopus*^14^, the 22 *Rhizopus* strains determined in this study belonged to three species: *R. microsporus* (three strains)*, R. delemar* (14 strains), and *R. arrhizus* (five strains) (Table 1). Based on ITS rRNA sequence data, the identities of many *Rhizopus* strains isolated from tempeh were rectified (Table 1). For example, *R. arrhizus* was changed to *R. delemar, R. oryzae* to *R. arrhizus, R. cohnii* to *R. arrhizus, R. microsporus* to *R. delemar, R. oryzae* to *R. delemar*, and *R. oligosporus* to *R. delemar.*

All *Rhizopus* strains isolated from tempeh belonged to three major groups in the phylogenetic tree: *R. arrhizus, R. delemar,* and the *R. microsporus* complex (Fig. 1). The resolution of sequences from the ITS regions was sufficient for identifying these *Rhizopus* species. The tree topology as seen in Fig. 1 was in agreement with the molecular taxonomic studies of *Rhizopus*^13,17,37^ and generally congruent with the tree topology based on phylogenomic approaches as inferred from a dataset of 192 orthologous genes^14^. The tree in Fig. 1 clearly indicates that *R. delemar* is a sibling or cryptic species of *R. arrhizus* and that they are very closely related, as evidenced by a 95% bootstrap value. The results from morphological characterizations (Fig. 2) also confirmed the results from molecular identification that all *Rhizopus* strains isolated from tempeh belong to *R. arrhizus, R. delemar,* and the *R. microsporus.*

Our group member (I.G.), isolated at least five species, namely *R. arrhizus*, *R. cohnii*, *R. microsporus*, and *R. stolonifer* from various tempeh products in Indonesia (mainly Java) in the 1960s–2000s. Previous identification of 127 *Rhizopus* strains isolated from tempeh based on morphological and physiological data revealed that *R. arrhizus* and *R. microsporus* were the most common species isolated from various tempeh products in Java in the 1960s–1970s (data not shown). These species identification results are in agreement with the findings of Hesseltine^23^, who reported *Rhizopus* species that were commonly used to produce tempeh: *R. arrhizus* and *R. microsporus*.

The results of molecular identification (Table 1) and the phylogenetic tree based on the ITS regions in the rRNA gene sequence data of *Rhizopus* strains isolated from tempeh before the use of commercialized tempeh starter revealed that the strains belonged to three species: *R. delemar*, *R. arrhizus,* and *R. microsporus* (Fig. 1)*.* It is clear that both *R. delemar* and *R. arrhizus* are the most commonly isolated *Rhizopus* species from tempeh in Java. The identity of tempeh molds verified in the present is in accordance with the findings of Arbianto^38^, who reported that two *Rhizopus* species are involved in the traditional process for tempeh production. In this process, *R. arrhizus* are a strong amylase, protease, and pectinase producers. After the temperature increases, *R. microsporus*, which can better withstand higher temperatures (37–40 °C), completes the process. Another study, Samson et al*.*^39^ also reported that *R. arrhizus* and *R. microsporus* were the most commonly isolated *Rhizopus* species from 110 commercial tempeh products in the Netherlands. Tempeh was introduced to the Netherlands by immigrants from Indonesia^3^.

As shown in Fig. 1, two species, *R. arrhizus* and *R. delemar*, were most commonly species found in tempeh in Java in the 1960s–1970s and in other regions in Indonesia before the 2000s. These species were originated from the leaves of *Hibiscus tiliaceus*, the leaves that were used as traditional tempeh starter (*usar*) in that period in Indonesia. Nout et al*.*^40^ found that *R. arrhizus* and *R. microsporus* were the predominant epiphytic molds on *Hibiscus tiliaceus* leaves (*daun Waru*) in Indonesia.

In the 1960s, I. G. isolated many *Rhizopus* strains from tempeh “Malang” and tempeh “Purwokerto” and found that *R. arrhizus* to be dominant tempeh molds from tempeh “Malang” and tempeh “Purwokerto”. However, 30 years later (in the 1990s), on isolating *Rhizopus* species from tempeh “Malang” and tempeh “Purwokerto”, she found *R. microsporus* to be dominant^20^.

These days, *Rhizopus* species other than *R. microsporus* are rarely found in tempeh in Indonesia. Hartanti et al.^41^ reported that 35 out of 36 *Rhizopus* strains isolated from fresh tempeh from 26 locations (mainly in Java) in Indonesia in 2012 and 2013 belonged to *R. microsporus*. Only one isolate from Sulawesi belonged to *R. delemar*. In a recent study on the genetic diversity of *Rhizopus* species isolated from the traditional inoculum of tempeh (*daun Waru*) all isolates were found to belong to a single species, *R. microsporus*^42^. In other surveys^43,44^, tempeh producers in Indonesia generally do not use their own traditional starters anymore. They use commercial starters, such as *Raprima* which can be purchased online and sold abroad. The use of commercial of tempeh starters is not limited to Java; it has spread to other regions in Indonesia since the 2000s.

Interestingly, Anggriawan^42^ performed RAPD typing of 471 pure *Rhizopus* isolates obtained from 247 samples of fresh tempeh and its inoculum from 16 provinces in Indonesia in 2013–2015 and found that *R. arrhizus, R. delemar,* and *R. stolonifer* were present in the samples collected outside Java, while the *R. microsporus* complex, was present in the samples collected within Java. These findings indicated that some tempeh producers outside Java still use the traditional process for tempeh production. Therefore, other *Rhizopus* species could be detected.

Sukardi et al.^45^ reported that the use of commercial tempeh starters containing *R. microsporus* is not suitable for tempeh production in Malang, East Java (which is located on a cool plateau). The *R. microsporus* inoculum results in the production of a less compact tempeh cake, which sometimes has an alcoholic smell. Moreover, the optimum growth temperature of *R. arrhizus* is lower than that of *R. microsporus*; therefore, *Rhizopus arrhizus* is more suitable for tempeh production in Malang.

Based on molecular evidence from ITS rRNA gene data, many strains validated in the present study had been misidentified (Table 1). The identification of *Rhizopus* species from tempeh based on morphological observation is not easy, especially within the *R. arrhizus* complex and *R. microsporus* complex. The high similarity in their morphological characteristics often leads to misidentification. *Rhizopus arrhizus* and *R. delemar* are sibling species and morphologically identical^10^. Despite the close genetic relationships between members of the *R. arrhizus* *sensu lato* and among the members of the *R. microsporus* complex, Zheng et al*.*^12^ mentioned in their monograph that these species have already undergone marked changes in their morphology while adapting themselves to their artificial environment, as fermentative agents over many generations. These morphological changes make the identification of these species very difficult based on their descriptions provided in a previous monograph by Schipper^8^. Therefore, Zheng et al*.*^12^ produced a monograph of *Rhizopus* based on the sporangial morphology, making it one of the most important references for the classification of the genus *Rhizopus*. Specifically, in the synoptic key to the classification of *Rhizopus*, sporangiophores exhibit simple, sometimes forked, branching at the upper portion and at the base and are rarely verticillate. Swelling is common, mostly at the middle or apical portion. As shown in Fig. 3, some *R. arrhizus, R. delemar,* and *R. microsporus* strains isolated from tempeh exhibited the branching and swelling of sporangiophores at the middle and upper portions. Multiple sporangia were observed at the upper or apical portion of sporangiophores and were verticillate.

In the present study, using a light microscope, we identified unique characteristics in some *Rhizopus* strains isolated from tempeh; these included the presence of sporangiophores with more than two branches or a single sporangiophore with more than two sporangia (multiple sporangia). Multiple sporangia were observed in *R. arrhizus, R. delemar,* and *R. microsporus* (Fig. 3). Similarly, Jennessen et al.^46^ reported double sporangia in *R. microsporus* CBS 112.586; however, a case of multiple sporangia has never been reported in this species. Our findings confirmed that the morphological changes in *Rhizopus* species isolated from tempeh as a result of centuries of domestication. Zheng et al*.*^12^ reported morphological changes in other *Rhizopus* species of economic importance, such as *R. microsporus* and *R. stolonifer.*

The present study verified the identity of *Rhizopus* strains used 40–50 years ago to produce tempeh using the traditional process in Indonesia. Phylogenetic analyses revealed that *R. arrhizus* and *R. delemar* were commonly found in various locations in Java 40–50 years ago (Table 1). However, neither species is found today because of the widespread use of the commercial tempeh starter *Ragi Raprima*® containing only *R. microsporus* in Indonesia.

The loss of genetic diversity of *Rhizopus* species in tempeh has changed the taste and flavor of tempeh. We do feel the impact of using commercialized inoculum in Java. Good–quality tempeh “Malang” and tempeh “Purwokerto”, which contain *R. arrhizus* complex, cannot be found anymore. The white wooly appearance and pleasant aroma of the famous tempeh Malang and tempeh Purwokerto have been replaced by plain white tempeh, because the aforementioned species have been replaced by *R. microsporus*, which is present in the commercial inoculum. Fortunately, the precious *Rhizopus* strains that were isolated from tempeh Malang in the 1960s–1970s are still preserved in UICC.

In summary, the ITS regions of the rRNA gene sequence data and phylogenetic analyses confirmed that the *Rhizopus* strains associated with tempeh fermentation using traditional inocula in Indonesia belong to three species: *R. arrhizus, R*. *delemar*, and *R. microsporus.* The wide use of commercial tempeh starters containing a single species, *R. microsporus* in Java has decreased the genetic diversity of *Rhizopus* species in tempeh and reduced the quality of tempeh Malang. The heavy commercialization of these tempeh starters has thus resulted in a change in the diversity of *Rhizopus* species associated with tempeh in Java in the last 30 years (since the 1990s).

Our findings confirmed the loss of *Rhizopus* diversity in tempeh currently produced in Indonesia, particularly in Java, where tempeh originated. We concluded that *R. delemar* and *R. arrhizus* have been lost from tempeh in Java. The present study makes an important contribution to validating the diversity of *Rhizopus* species, which were commonly used for tempeh production in Indonesia in the past (before the use of commercial tempeh starters). These strains have been securely deposited in a culture collection in Indonesia, and their sequence data have been deposited in a sequence database. The present findings emphasize the importance of conserving the *Rhizopus* strains isolated from tempeh produced using the traditional process in the past in culture collections in order to preserve and restore our precious genetic resources for conservation and sustainable use.
